# The Omicron Sub-Variant BA.4 Displays a Remarkable Lack of Clinical Signs in a Golden Syrian Hamster Model of SARS-CoV-2 Infection

**DOI:** 10.3390/v15051133

**Published:** 2023-05-10

**Authors:** Elizabeth R. Davies, Kathryn A. Ryan, Kevin R. Bewley, Naomi S. Coombes, Francisco J. Salguero, Oliver T. Carnell, Sarah Biddlecombe, Michael Charlton, Amy Challis, Eleanor S. Cross, Alastair Handley, Didier Ngabo, Thomas M. Weldon, Yper Hall, Simon G. P. Funnell

**Affiliations:** 1 UKHSA Porton, Vaccine Development and Evaluation Centre, UK Health Security Agency, Manor Farm Road, Salisbury SP4 0JG, UK; 2 Quadram Institute Bioscience, Norwich Research Park, Norwich NR4 7UQ, UK; 3 World Health Organization, Appia 20, 1211 Geneva, Switzerland

**Keywords:** SARS-CoV-2, Syrian hamster, animal model, coronavirus

## Abstract

The ongoing emergence of SARS-CoV-2 virus variants remains a source of concern because it is accompanied by the potential for increased virulence as well as evasion of immunity. Here we show that, although having an almost identical spike gene sequence as another Omicron variant (BA.5.2.1), a BA.4 isolate lacked all the typical disease characteristics of other isolates seen in the Golden Syrian hamster model despite replicating almost as effectively. Animals infected with BA.4 had similar viral shedding profiles to those seen with BA.5.2.1 (up to day 6 post-infection), but they all failed to lose weight or present with any other significant clinical signs. We hypothesize that this lack of detectable signs of disease during infection with BA.4 was due to a small (nine nucleotide) deletion (∆686–694) in the viral genome (ORF1ab) responsible for the production of non-structural protein 1, which resulted in the loss of three amino acids (aa 141–143).

## 1. Introduction

The history of the SARS-CoV-2 pandemic has been associated with an unprecedented level of global genomic surveillance. This surveillance has been fundamental for the identification and monitoring of virus variants as they have continued to emerge. On November 26, 2021, WHO designated Omicron (B.1.1.529) a variant of concern [1]. Omicron became the dominant SARS-CoV-2 variant globally due to increased transmissibility and the ability to evade both natural and vaccine-induced immunity [2,3,4].

While different animal models of SARS-CoV-2, including the ferret [5,6,7,8,9] and non-human primate [10,11,12], have been established, the Golden Syrian hamster *(Mesocricetus auratus)* model is best established as a model of SARS-CoV-2, exhibiting signs of severe clinical disease [13] with the protype virus. Several studies have shown that Omicron presents as a less severe disease in the hamster model [14,15,16]. Armando et al. (2022) have shown that Omicron presented as a sub-clinical infection in hamsters, characterized by a low viral load, mild pathology, and decreased inflammatory cell infiltrates in the nasal cavity and lungs at 4 days post-infection [16]. Yuan et al. (2022) demonstrated something similar but also demonstrated that in vaccinated hamsters, Omicron could outcompete Delta and could transmit more effectively to co-housed companions [15]. The reduced pathogenicity demonstrated in the hamster model mirrors that seen in the human population. Although most people now have some degree of protection, either from vaccine-induced immunity, prior infection, or both, the rapid ability for Omicron to spread regardless of immune status continues to be a concern. Therefore, the hamster model remains an important tool for delineating the pathogenic potential of new emerging variants of concern.

Omicron has continued to evolve and has developed distinct sub-lineages, one of which is BA.2 [17]. Sub-lineage BA.2 has continued to evolve, and its phylogenetic “offspring” include BA.4 and BA.5.2.1, which were first described in South Africa in early 2022 [18]. The sub-lineages BA.4 and BA.5.2.1 have a spike protein sequence comparable to BA.2 but have additional mutations resulting in several amino acid changes: a deletion 69–70 and substitutions L452R, F486V, and R493Q [18,19]. Sub-lineage BA.4 used in this study has an additional non-defining amino acid substitution in the spike (S640F). Other mutations result in substitutions in nucleoproteins (N:P151S) and Orf7b (L11F). BA.4 has a deletion not found in other sub-lineages in Orf1a, resulting in the loss of three amino acids; Nsp1: Δ141–143 [19]. Evidence suggests that existing COVID-19 ancestral spike-based vaccinations may be less efficacious at producing neutralizing antibodies to BA.4 and BA.5.2.1 [20]. This study sought to investigate potential differences in pathogenicity between these sub-lineages and previous circulating Omicrons.

## 2. Materials and Methods

**Viruses.** The GISAID ID or source of the isolation swabs/viruses used in this study is as follows: Ancestral (EPI_ISL_406844, [21]), BA.1 (EPI_ISL_7400555), BA.2 (not available), BA.2.12.1 (Gavin Screaton, University of Oxford, Oxford, UK), BA.4 (EPI_ISL_13157810), BA.5.2.1 (EPI_ISL_12810908), BQ.1.22 (EPI_ISL_15581064), XBB.1.1 (EPI_ISL_15682231). Viruses were isolated and propagated on Vero/hSLAM (ECACC 04091501, European Collection of Authenticated Cell Cultures (ECACC) UKHSA, Porton Down, UK), with quality control checks and whole genome sequencing performed as previously described [22]. Sequence data for the virus banks used in this study is available in Supplementary Data File S1.

**Animals.** Twenty-four healthy Golden Syrian hamsters (*Mesocricetus auratus*), aged 7–12 weeks, were obtained from a UK Home Office-accredited supplier (Envigo RMS UK, Oxon, UK). Animals were housed individually at Advisory Committee on Dangerous Pathogens (ACDP) containment level 3. Cages met the UK Home Office Code of Practice for the Housing and Care of Animals Bred, Supplied, or Used for Scientific Procedures (December 2014). Access to food and water was ad libitum, and environmental enrichment was provided. All experimental work was conducted under the authority of a UK Home Office project license that had been subject to local ethical review at UKHSA Porton Down by the Animal Welfare and Ethical Review Body (AWERB) as required by the Home Office Animals (Scientific Procedures) Act 1986.

**Study Design.** Hamsters (*n* = 6 per group, equal male to female ratio) were randomly assigned to groups to minimize bias using the RAND() function in Excel. A biothermal identifier chip (Plexx IPTT-300 temperature transponder) was inserted subcutaneously into each animal under sedation. Prior to infection, animals were sedated with isoflurane. The virus was delivered by intranasal instillation (200 μL total, 100 μL per nare) diluted in phosphate buffered saline (PBS). Groups 1 and 2 were infected with SARS-CoV-2 Omicron BA.4 for a target dose of 1.0 × 10^4^ (FFU). Groups 3 and 4 were infected identically, but with the sub-variant BA.5.2.1. Groups 1 and 3 were taken for necropsy 7 days after infection, and groups 2 and 4 were taken at day 28. Hamsters were throat swabbed at days −2, 2, 4, 6, 8, 10, 14, and 21 post-infection, as well as directly before necropsy (in-life). Blood samples were collected at baseline and necropsy to measure the humoral immune response.

**Clinical observations.** Hamsters were monitored twice daily (approximately 8 h apart) to record temperature via biothermal Identichip and clinical signs of disease. Clinical signs of disease were assigned based upon the following criteria: healthy, lethargy, behavioral change, sunken eyes, ruffled, wasp-waist, dehydrated, arched, coughing, and labored breathing (1—occasional catch or skip in breathing rate and labored breathing; 2—abdominal effort with breathing difficulties). The prevalence of each sign produced a weighted score at each time point. Clinical signs not observed have not been presented. Animals were weighed at the same time each day until the scheduled necropsy.

**Necropsy Procedures.** Hamsters were given an anesthetic overdose (sodium pentabarbitone Dolelethal, Vetquinol UK Ltd., Titchmarsh, UK, 140 mg/kg) via intraperitoneal injection, and exsanguination was by cardiac puncture. A necropsy was performed immediately after the confirmation of death.

**RNA Extraction.** Throat swabs were inactivated in AVL (Qiagen, UK) and ethanol, and RNA was isolated. Downstream extraction was performed using the BioSprint™96 One-For-All Vet kit (Indical, Leipzig, Germany) and Kingfisher Flex platform as per the manufacturer’s instructions.

**Quantification of Viral RNA by RT-qPCR.** Reverse transcription-quantitative polymerase chain reaction (RT-qPCR) targeting a region of the SARS-CoV-2 nucleocapsid (N) gene was used to determine viral loads and was performed using TaqPathTM 1-Step RT-qPCR Master Mix, CG (Applied Biosystems, Waltham, MA, USA), the 2019-nCoV CDC RUO Kit (Integrated DNA Technologies, Coralville, IA, USA), and the QuantStudio 7 Flex Real-Time PCR System (Thermofisher, Loughborough, UK). N1 primers and probe sequence: 2019-nCoV_N1-forward, 5′ GACCCCAAAATCAGCGAAAT 3′; 2019-nCoV_N1-reverse, 5′ TCTGGTTACTGCCAGTTGAATCTG 3′; 2019-nCoV-N1-probe, 5′ FAM-ACCCCGCATTACGTTTGGTGGACC-BHQ1 3′. Cycling conditions were: 25 °C for 2 min, 50 °C for 15 min, 95 °C for 2 min, followed by 45 cycles of 95 °C for 3 s, and 55 °C for 30 s. The quantification standard was in vitro transcribed RNA of the SARS-CoV-2 N ORF (accession number NC_045512.2) with quantification between 1.0 × 10^1^ and 1.0 × 10^6^ copies/μL. Positive swab and fluid samples detected below the limit of quantification (LLOQ) of 12,857 copies/ mL were assigned a value of 5 copies/μL; this equates to 6429 copies/mL, whereas undetected samples were assigned a value of <2.3 copies/μL, equivalent to the assay’s lower limit of detection (LLOD), which equates to 2957 copies/mL.

**Focus-forming assay (FFA).** The viral titer of infection material and throat swabs (TS) was determined by a focus-forming assay on Vero/E6 cells [ECACC 85020206]. Cell lines were obtained from the European Collection of Authenticated Cell Cultures (ECACC) at the UKHSA, Porton Down, UK. 96-well plates were seeded with 2.5 × 10^4^ cells/well the day before infection, then washed twice with Dulbecco’s PBS (DPBS). Ten-fold serial dilutions (1.0 × 10^−1^ to 1.0 × 10^−6^) of virus stocks were prepared in serum-free MEM (supplemented with 25 mM HEPES (Gibco, Paisley, UK), 2 mM L-Glutamine (Gibco), 1× Non-Essential Amino Acid Solution (Gibco), 1× antibiotic, antimycotic (Gibco). A 100 μL sample of virus inoculum was added to each well in duplicate and incubated at 37 °C for 1 h. Virus inoculum was removed, and cells were overlaid with MEM containing 1% carboxymethylcellulose (Sigma, Hertforshire, UK), 4% (*v*/*v*) heat-inactivated fetal bovine serum (FBS) (Sigma), 25 mM HEPES buffer, 2mM L-Glutamine, 1x Non-Essential Amino Acid Solution, 1× antibiotic, and 1× antimycotic. After incubation at 37 °C for 22 h (BA.5.2.1) or 26 h (BA.4), cells were fixed overnight with 20% formalin/PBS, with immuostaining as described previously [23] with modifications for Omicron: After cells were fixed, and prior to the removal of residual endogenous peroxidase activity with 0.3% hydrogen peroxide (Sigma, UK), cell monolayers were permeabilized with 0.2% Triton-X-100/PBS. Cells were incubated for 1 h with a primary rabbit polyclonal antibody against SARS-CoV-2 anti-nucleocapsid, diluted 1:1000 (Sinobiological, Beijing, China, 40588-T62), before resumption of the previously described protocol [23]. Both primary and secondary antibodies were diluted in 0.2% Triton-X-100/PBS. Titer (FFU/mL) was determined by the following formula: Titer (FFU/mL) = No. of foci/(dilution factor × 0.1).

**SARS-CoV-2 focus reduction neutralization test.** Hamster sera were heat-inactivated at 56 °C for 30 min to destroy any complement activity and serially diluted 1:2 in MEM containing 1% fetal calf serum, 1% L-Glutamine, 25 mM HEPES, and 1% antibiotic-antimycotic. The virus was diluted to give 100–250 foci in the virus-only controls and added to the serum dilutions before incubation for 1 h at 37 °C. Serum/virus mixtures were incubated on a VeroE6 cell monolayer (ECACC) for 1 h at 37 °C. The virus/antibody mixture was replaced with overlying media containing 1% CMC before being incubated overnight for 22 (BA.5.2.1), 24 (VIC01), or 26 h (BA.1, BA.4, BQ.1.22, XBB.1.1) at 37 °C. Cells were fixed overnight by adding 20% formalin/PBS solution. Immunostaining for VIC01 was performed as described previously [23]. The modifications described in this paper were used for immunostaining BA.1, BA.4, BA.5.2.1, BQ.1.22, and XBB.1.1 and are described in the focus formation assay.

**Histopathology.** The following samples from each animal were fixed in 10% neutral-buffered formalin, processed to paraffin wax, and 4 µm-thick sections cut and stained with hematoxylin and eosin (H&E): nasal cavity and respiratory tract (left lung lobe). Tissue sections were scanned using a Hamamatsu S360 scanner and viewed by ndp.view2 software (Hamamatsu, Shizuoka, Japan, U12388-01). Tissue sections were evaluated subjectively by a veterinary pathologist blinded to treatment and group details, and the slides were randomized prior to examination to prevent bias. A scoring system previously reported by our group was applied independently to the lung tissue sections and nasal cavity [24]. The severity of the pulmonary microscopic lesion was also evaluated using digital image analysis (Nikon-NIS Br) to calculate the percentage area of pneumonia in H&E-stained lung tissue sections. RNAscope (an in-situ hybridization method used on formalin-fixed, paraffin-embedded tissues) was used to identify the SARS-CoV-2 virus in all tissues. Briefly, tissues were pre-treated with hydrogen peroxide at room temperature for 10 min, target retrieval for 15 min (98–101 °C), and protease plus for 30 min (40 °C) (all Advanced Cell Diagnostics, Abingdon, UK). A V-nCoV2019-S probe (Advanced Cell Diagnostics) targeting the S-protein gene was incubated on the tissues for 2 h at 40 °C. Amplification of the signal was carried out as per the manufacturer’s instructions using the RNAscope 2.5 HD red kit (Advanced Cell Diagnostics). RNAScope-stained sections were also scanned, and digital image analysis was carried out to calculate the total area of the lung section positive for viral RNA. For the nasal cavity, a semiquantitative scoring system was applied to evaluate the presence of virus RNA: 0 = no positive staining; 1 = minimal; 2 = mild; 3 = moderate; and 4 = abundant staining.

**Statistics.** The normal distribution of the numeric data was evaluated, and appropriate parametric or non-parametric statistical tests were applied. Weight percentage change data was analyzed by a Tukey-corrected pairwise multiple comparison ANOVA. Parametric statistical analyses were selected as the data were expected to conform to a log-normal distribution (for qPCR results) or a normal distribution (for weights and antibody titers) based on historical observations of data from similar hamster challenge studies. Histopathological results were analyzed by Mann-Whitney’s U test. Paired *t*-tests were used to analyze variant differences in convalescent hamster sera.

## 3. Results

**Study design.** Hamsters (*n* = 12 per group, with equal numbers of males and females) were infected intranasally with either BA.4 or BA.5.2.1 to achieve a target dose of 1.0 × 10^4^ focus-forming units (FFU) in a total volume of 200 μL (100 μL per nare) per hamster. Inocula were back-titrated by focus-forming assay (FFA) [9] on the day of infection to confirm doses. Throat swabs (TS) were taken at −2, 2, 4, 6, 8, 10, 14, 21, and 28 days post-infection (DPI). Six hamsters infected with BA.4 and six infected with BA.5.2.1 were culled on day seven post-infection. The remaining hamsters were culled on day 28 post-infection. Viral loads in the lung were determined at days 7 and 28 post-infection. Pathology was assessed in the nasal turbinates and lungs. Sera were taken at baseline and cull (either 7 or 28 days post-infection) for analysis of the humoral response.

**Figure 1:**
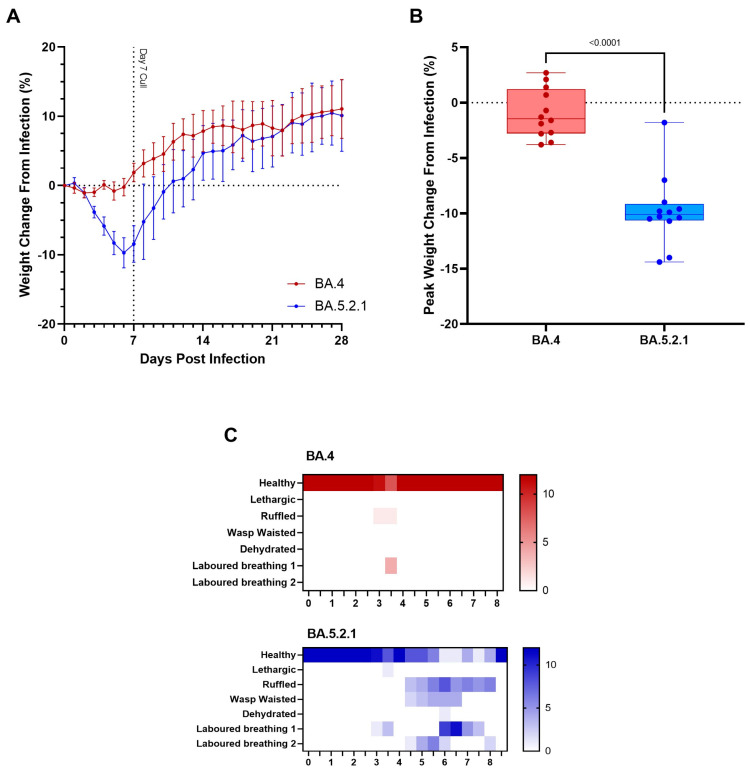
Clinical signs in Omicron Variants BA.4 and BA.5.2.1 infected hamsters. Hamsters were monitored for (**A**) weight loss following infection with Omicron variants BA.4 or BA.5.2.1. Lines show group means; error bars represent 95% confidence interval. (**B**) Peak weight change at 6 days post infection as a percentage of the initial weight at the day of infection. Box plots show medians and 25th to 75th percentiles, and whiskers represent minimum and maximum values; all data points are shown. Statistical analyses for peak weight change were performed using Mann-Whitney Test. (**C**) Number of hamsters displaying each clinical sign at each observation point.

**BA.5-infected animals exhibit weight loss**. In our Golden Syrian Hamster model of SARS-CoV-2 infection [25] and others [13], weight loss is typically one of the first clinical signs of infection observed (1–2 days post-infection). Hamsters were weighed daily, and a percentage weight change was calculated from the day of infection. Animals that were infected with BA.4 ceased to gain weight in the first few days following infection (Figure 1A, red line); however, from the first day following infection onwards, the animals infected with BA.5.2.1 demonstrated prolonged weight loss until 7 days post-infection (Figure 1A, blue line). Weight change was significant between groups from 3 DPI (*p* < 0.0001) until 13 DPI (*p* = 0.0426), with peak weight loss observed at 6 DPI (*p* < 0.0001) in the BA.5.2.1-infected hamsters (Figure 1B). As other clinical signs of infection subsided, BA.5.2.1-infected animals began to regain weight and were back to their pre-infection weights by 11 days post-infection. From that timepoint, their weights were comparable to the weights observed in the BA.4-infected hamsters.

**BA.5-infected animals exhibit increased clinical signs.** As required by our UK Home Office project license, clinical signs of infection were monitored and recorded twice a day following the challenge. Except for 3 days post-infection, few clinical signs were seen in BA.4-infected hamsters; labored breathing was observed in 4/12 and ruffled fur in 1/12 at day 3 post-infection at one monitoring point only (Figure 1C, red heatmap). In contrast, multiple clinical signs were seen in all hamsters infected with BA.5.2.1 over several sequential days (Figure 1C, blue heatmap); these were most frequently ruffled fur, wasp-waist appearance, and labored breathing (where a score of 1 is ‘evident’ and 2 is ‘pronounced’).

**Figure 2:**
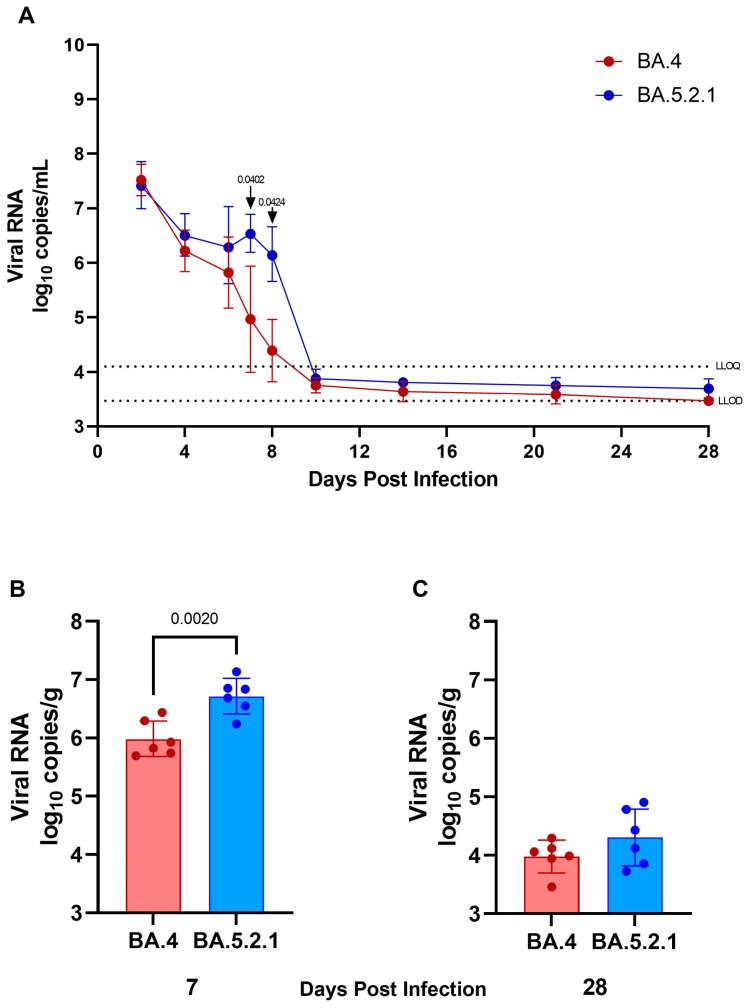
Viral shedding from the upper respiratory tract and viral load in the lung. To quantify viral shedding and viral load, throat swabs and lung tissue were taken, and RNA isolated from inactivated samples. (**A**) Viral shedding from the upper respiratory tract, viral load in the lung at 7 (**B**) and 28 days (**C**) post infection. Graphs shows geometric mean and SD. Statistical analyses were performed using unpaired *t* test with Welch’s correction on log_10_ transformed data (*p* < 0.05 displayed).

**Viral shedding and lung viral loads are reduced in BA.4-infected hamsters.** Viral shedding was determined by RT-qPCR targeting a region of the SARS-CoV-2 nucleocapsid (N) gene. At 2 and 4 days post-infection, similar levels of viral shedding were seen in all hamsters, regardless of which Omicron virus was administered by the intranasal route (Figure 2A). Assessment of live virus load by FFA in throat swabs at 2 days post-infection also confirmed no difference in live viral titer (Figure S1). From 6 days post-infection onward, reduced viral shedding was observed in BA.4-infected hamsters. The difference in shedding from the upper respiratory tract in BA.4- and BA.5.2.1-infected hamsters was found to be significant at both 7 and 8 days post-infection (*p* = 0.0402 and 0.0424, respectively). From day 10 onward, levels of viral shedding fell below the lower levels of quantification and detection for all infected hamsters. At cull, in lung tissue from hamsters infected with BA.5.2.1 for 7 days, there was significantly more viral load compared to BA.4-infected hamsters (Figure 2B) (*p* = 0.002); however, at cull 28 days post-infection, there was no difference, and the viral load was observed to be below the lower limit of quantification (Figure 2C).

**Figure 3:**
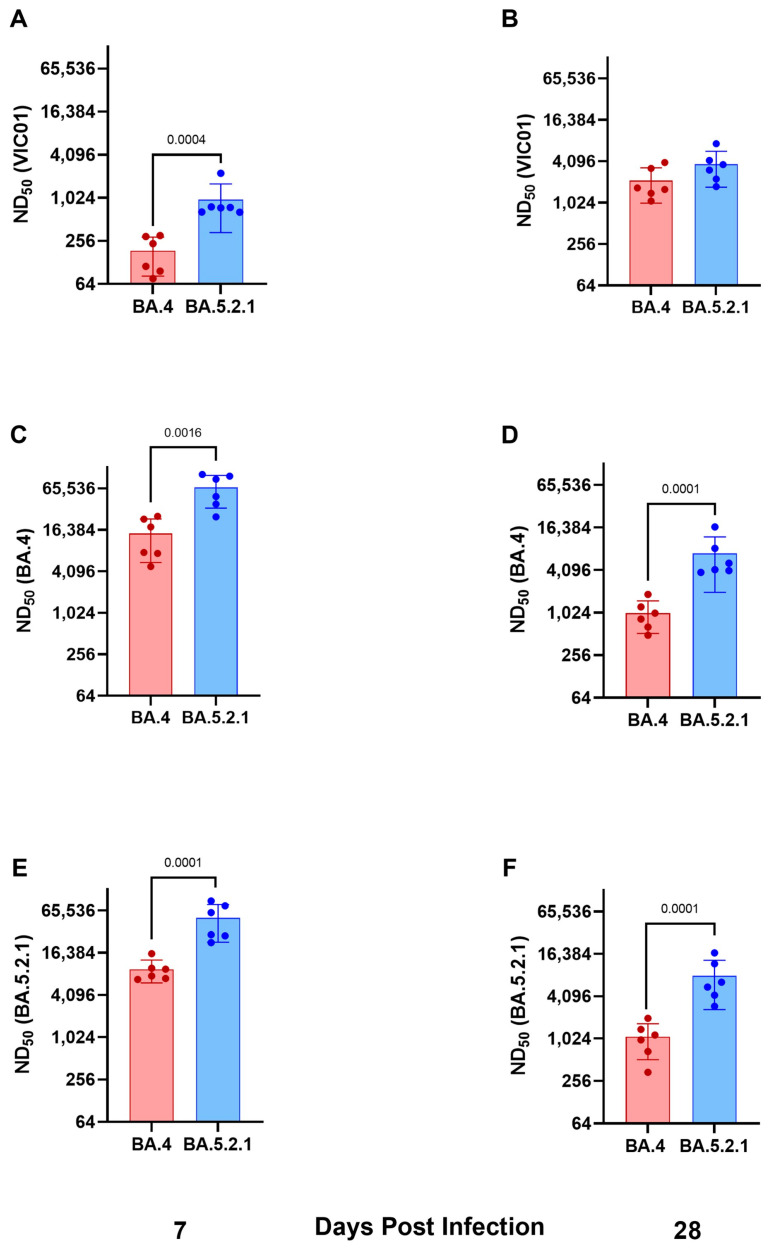
Humoral response to ancestral virus and Omicron sub variants BA.4 and BA.5.2.1. Neutralization assay with heat inactivated sera to determine neutralizing antibody titers in the infected hamsters to (**A**,**B**) Victoria/1/2020, (**C**,**D**) BA.4 and (**E**,**F**) BA.5.2.1 at 7- and 28- days post-infection respectively. Bar graphs show geometric mean and SD; all data points are shown. Statistical analyses were performed using unpaired *t* test with Welch’s correction on log_10_ transformed data (*p* < 0.05 displayed).

**Convalescent hamsters infected with BA.4 have lower neutralizing antibody titers against ancestral wild-type viruses and Omicron variants.** Geometric neutralizing antibody titers (ND50) in hamster sera were determined in samples taken at 7 and 28 days post-infection against ancestral viruses (Australia/VIC01/2020, VIC01), BA.4, and BA.5.2.1. Neutralizing antibody responses were detected in all hamsters against ancestral virus and both Omicron sub-variants regardless of challenge virus; however, hamsters infected with BA.5.2.1 had higher titers against ancestral, BA.4, and BA.5.2.1 at both timepoints. Geometric mean neutralizing titers are shown in Table 1. Figure 3A,B illustrate neutralizing antibodies to VIC01; Figure 3C,D to BA.4; and Figure 3E,F to BA.5.2.1. Live virus neutralizing antibody titers against BA.4 and BA.5.2.1 fell between 7 and 28 days post-infection, in contrast to the increase over the same time period for the titers detected against the ancestral virus. We also noted that hamsters infected with BA.5.2.1 elicited higher live virus neutralizing antibody titers against BA.4 than hamsters infected with BA.4.

**Figure 4:**
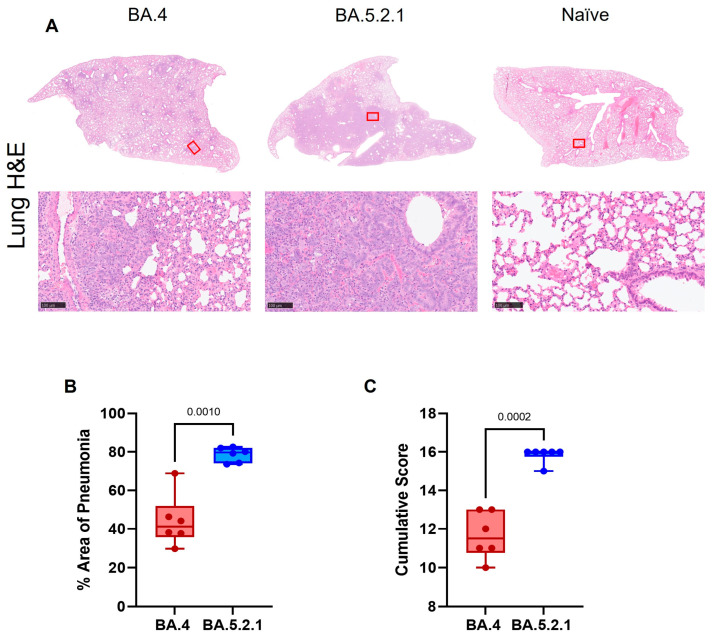
Histopathological analysis of lung tissue 7 days post infection. Lungs were fixed in 10% neutral-buffered formalin, processed to paraffin wax and 4 µm thick sections cut and stained with H&E. (**A**) Representative images of H&E stained lung of BA.4 (**left** panels), BA.5.2.1 (**center** panels) and naïve control (**right** panels) hamsters (bar represents 100 µm). The red box indicates the area of higher magnification. (**B**) Percentage of areas of pneumonia in the lung and (**C**) cumulative histopathology score, both determined by image analysis (Nikon-NIS-Ar). Box plots show medians and 25th to 75th percentiles, and whiskers represent minimum and maximum values; all data points are shown. Statistical analyses were performed using an unpaired *t* test with Welch’s correction.

**Pathology.** Histopathological examination showed multifocal areas of bronchointerstitial pneumonia in all hamsters at day 7 post-infection (Figure 4A, left and center panels). Representative images of mock-infected hamsters are included for comparison (where no lesions were observed). However, the severity of microscopic lesions, the area of the lung showing pneumonia (Figure 4B), and the total histopathology score (Figure 4C) of the lung were significantly lower in BA.4-infected hamsters when compared with BA.5.2.1-infected hamsters. At day 28 post-infection, hamsters showed less severe lung lesions, with type II pneumocyte hyperplasia and low-grade peribronchiolar and perivascular cuffing as the main observed changes in the lung. Viral RNA (detected by in situ hybridization of the -S-gene region) was seen in the lung of only four hamsters (two infected with BA.4; two infected with BA.5.2.1) at day 7 post-infection. No viral RNA was detected in any hamsters at day 28 post-infection in the lung.

**Figure 5:**
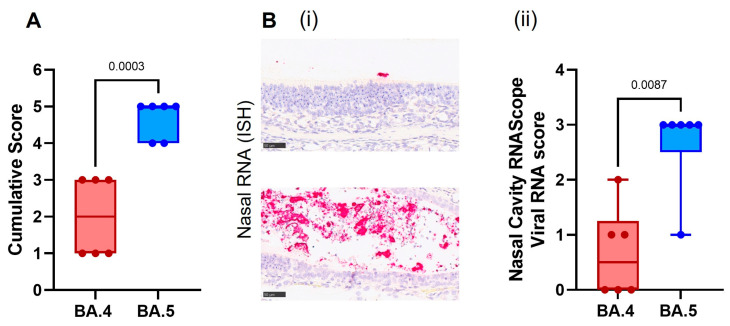
Histopathological findings in the nasal cavity of BA.4 or BA.5.2.1 infected hamsters 7 days post infection. Heads were fixed in 10% neutral-buffered formalin and the nasal cavity stained with H&E. (**A**) A blinded scoring system was used to compare the severity of the nasal cavity for each individual animal and among groups. (**B**, **i**) Representative images of SARS-CoV-2 RNA in the nasal cavity measured by in situ-hybridization (ISH) (bar represents 50µm). (**B**, **ii**) Subjective scores of presence of viral RNA in the nasal cavity. Box plots show medians and 25th to 75th percentiles, and whiskers represent minimum and maximum values; all data points are shown. Statistical analyses were performed using unpaired *t* test with Welch’s correction.

Cell necrosis was observed in the respiratory and olfactory epithelium associated with inflammatory exudates within the nasal cavity laminae and the presence of viral RNA at day 7 post-infection, showing significantly less pathology (Figure 5A) and viral RNA (Figure 5B) in the epithelial cells and exudates in the BA.4-infected hamsters compared to the BA.5.2.1-infected hamsters. Only minimal microscopic changes and no viral RNA were observed in the nasal cavity at day 28 post-infection.

**Figure 6:**
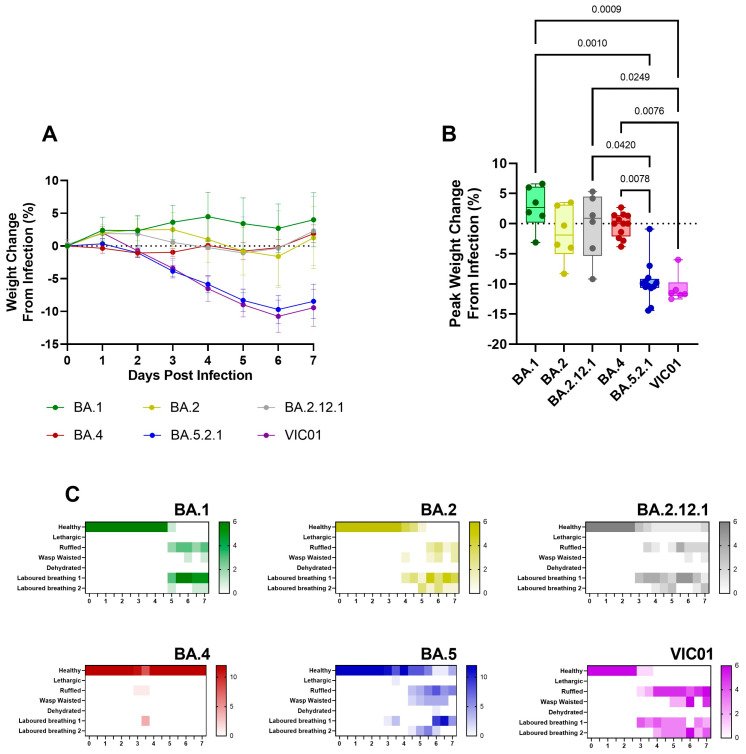
Comparison of weight loss and clinical signs in earlier Omicron sub-lineages BA.1, BA.2, BA.2.12.1 and Ancestral VIC01. Hamsters were monitored for (**A**) weight loss following infection. Lines show group means; error bars represent 95% confidence interval. (**B**) Peak weight change at 6 days post infection as a percentage of the initial weight at the day of infection. Box plots show medians and 25th to 75th percentiles, and whiskers represent minimum and maximum values; all data points are shown. Statistical analyses for peak weight change were performed using one-way ANOVA (Kruskal-Wallis). (**C**) Number of hamsters displaying each clinical sign per observation point.

**Comparison to earlier Omicron variants.** Given the reports in the literature that show Omicron sub-lineages have thus far presented with sub-clinical disease [15,16,17], the stark comparison between BA.4 (following the existing Omicron sub-lineage trend) and the return of a more pathogenic phenotype seen with BA.5.2.1 was of interest. We therefore performed direct comparisons with data generated from our previous studies with the earlier Omicron sub-lineages in parallel with the ancestral virus. Consistent and prolonged weight loss was only seen in hamsters infected with BA.5.2.1 and ancestral virus; a lack of weight gain in the days following infection was observed with BA.1, BA.2, BA.2.12.1, and BA.4 (Figure 6A). Peak weight loss as measured at 6 days post-infection (Figure 6B) was significantly higher in BA.5.2.1 hamsters compared to BA.1 (*p* = 0.001), BA.2 (*p* = 0.042), and BA.4 (*p* = 0.0078), as well as in VIC01-infected hamsters compared to BA.1 (*p* = 0.0009), BA.2.12.1 (*p* = 0.0249), and BA.4 (*p* = 0.0076). Clinical signs of infection were observed for all other Omicron variants except for hamsters infected with BA.4. The incidence of clinical signs in BA.1, BA.2, and BA.2.12.1-infected hamsters was typically observed around 24 h after the onset in BA.5.2.1- or ancestrally-infected hamsters (Figure 6C). Viral shedding from the upper respiratory tract was approximately ten-fold lower in hamsters infected with BA.1, BA.2, and BA.2.12.1 when compared to ancestral and BA.5.2.1 isolates for the 7 days following infection (Figure S2A); however, there was no statistical difference in viral load in the lung between Omicron variants, only between ancestral virus and BA.1, BA.2.12.1, and BA.4, where *p* = 0.0244, 0.0102, and 0.0007, respectively (Figure S2B). This was also true for viral RNA (detected by in situ hybridization with the -S-gene), although this lack of statistical difference is likely due to an outlier in the BA.5.2.1-infected hamsters (Figure S3A). Furthermore, histopathological analyses demonstrated that areas of the lung showing microscopic lesions were significantly higher in BA.5.2.1- and ancestrally-infected hamsters as well as BA.2-infected hamsters (Figure S3B–E). Collectively, data show that BA.5.2.1 suggests a return to ancestral virus pathology in the Golden Syrian hamster model relative to the previously circulating Omicron variants, while BA.4 shows a notable lack of clinical signs despite only a few genetic changes.

**Figure 7:**
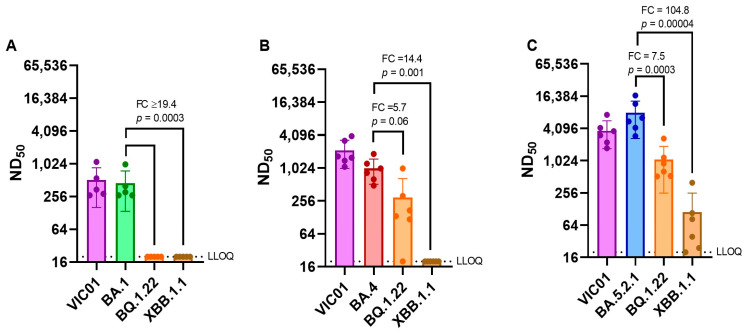
Comparison of the live neutralization titers of convalescent hamster sera previously infected with BA.1, BA.4 or BA.5.2.1 virus against emerging Omicron sub-lineages BQ.1.22 and XBB.1.1. 28 DPI hamster sera was tested against two of the more recent variants BQ.1.22 and XBB.1.1 for neutralization capability. Comparative neutralizing antibody titer to (**A**) BA.1 and (**B**) BA.4 and (**C**) BA.5.2.1. Bar graphs show geometric mean and SD; all data points are shown. Lower limit of quantification (LLOQ) is 20 (representing 1 in 20 starting dilution). Statistical analyses were performed using paired *t* test with Welch’s correction on log_10_ transformed data.

**Neutralizing antibody titers against emerging variants.** As SARS-CoV-2 continues to evolve and new variants emerge, we have assessed live virus neutralizing antibody levels in BA.1, BA.4, and BA.5.2.1 convalescent hamster sera (28 DPI) against more recent variants BQ.1.22 and XBB.1.1. The geometric mean neutralizing antibody titers of each group of hamsters are shown in Table 2. BA.1 convalescent sera demonstrated a ≥18-fold reduction in neutralization titer when we compared the homologous titer against BA.1 (373) with those detected against BQ.1.22 and XBB.1.1 (≤20). Both BA.4 and BA.5.2.1 convalescent sera also demonstrated a reduction in neutralizing titers for the emerging sub-lineages, but BA.5.2.1 infection appeared to induce better neutralizing antibody titers against all viruses tested. Figure 7 illustrates these differences in neutralizing antibody titers by comparing homologous titers with ancestral and Omicron sub-lineages.

## 4. Discussion

Genetic mutation and viral recombination have resulted in an “alphabet soup” of SARS-CoV-2 variants. The rapid emergence of some of these new variants constitutes a continued challenge to the success of the global vaccination program and controls directed at preventing continued transmission and associated morbidity and mortality.

In this study, we have demonstrated that there are two distinct disease profiles in hamsters after intranasal infection with either Omicron sub-variants BA.4 or BA.5.2.1, despite these viruses having almost identical spike proteins. Hamsters infected intranasally with BA.5.2.1 demonstrated significant weight loss (−9.8 ± 3.2%) by 7 DPI and multiple clinical signs of infection—comparable to those seen in animals infected with the ancestral virus (Australia/VIC01/2020) [9]. In contrast, hamsters infected with an equivalent virus dose of BA.4 maintained weight and displayed none of the clinical signs that have so far been seen in all the virulence studies we have conducted to date. Despite these clinical differences, viral shedding from the upper respiratory tract was similar for both Omicron variants in the days immediately following infection. We also assessed the impact of emerging variants BQ.1.22 and XBB.1.1 against convalescent hamster sera from BA.1-, BA.4-, and BA.5.2.1-infected hamsters, demonstrating a significantly reduced capacity to neutralize. While neutralization of BA.5.2.1 convalescent hamster sera against BQ.1.22 and XBB.1.1 appears to be enough to offer protection [14], BA.1 and BA.4 convalescent hamster sera demonstrated little to no neutralization against the same viruses. A likely explanation for this is the phylogeny of BQ.1.22 and XBB.1.1 [18,26] (offspring of BA.2) and the high titer of neutralizing antibodies observed in BA.5.2.1 convalescent hamsters.

We note the disease outcome observed here is different from the recent publication by Uraki et al. (2022), who describe no discernible differences between BA.4 and BA.5, where most notably there is no weight loss in hamsters infected with BA.5 [27]. While we use an infection inoculum volume of 200 µL [25], Uraki et al. used an infection inoculum of 30 µL. We postulate that the differences observed between the results we present and those of Uraki et al. could be due to the difference in inoculum volume or the additional changes in BA.5.2.1 used in this study. Our previously published data [25] demonstrate that SARS-CoV-2 disease severity in the Golden Syrian hamster model of infection is related to the volume of IN inoculum.

There are several possible explanations for the differences in pathogenicity observed in hamsters infected with either BA.4 or BA.5.2.1. Our hypothesis is that the deletion in the SARS-CoV-2 viral non-structural protein 1 (Nsp1) is the most likely. A nine-nucleotide deletion specific to BA.4 was identified in ORF1a (∆686–694), leading to the loss of three amino acids (aa 141–143) in Nsp1. In SARS-CoV-2, Nsp1 is likely to play many different key roles in the host cell [28]. Other previously published studies have reported that Nsp1 inhibits host protein translation and disrupts the mRNA export machinery to inhibit host gene expression [29,30,31]. In addition, Nsp1 of SARS-CoV was shown to cause the decay of host mRNA [32] and down-regulate type I IFN responses (IFN-α and IFN-β) [33,34].

Lin et al. [33] tracked the molecular evolution and clinical features of SARS-CoV-2-infected patients in China and showed that ∆500–532 in Nsp1 correlated with lower viral load, less severe symptoms of infection, and lower serum IFN-β. They also showed that IFN-I responses were significantly reduced in Calu-3 cells infected with deletion mutation viruses isolated from clinical samples or engineered using reverse genetics [33].

Fisher et al. (2022) have performed mutagenesis studies to demonstrate that Nsp1 is a major immune evasion factor in SARS-CoV-2 [35]. They generated a mutant SARS-CoV-2 with an amino acid deletion (aa 155–156) by reverse genetics and infected Vero E6 (which does not produce type I interferon) [36] and Calu-3 (which generates an intact interferon response) cells. They showed comparable viral titers in Vero-E6 cells, but the mutant viral titers were lower in the Calu-3 cells compared to wild-type viruses. They also demonstrated a stronger induction of interferon-stimulated genes in Calu-3 cells infected with mutant viruses to support Nsp1′s role in down-regulating the interferon response [35]. Deletions in related Coronaviruses have been shown to play an important part in their virulence [31,37,38]. Given the role of Nsp1 as a major pathogenic factor, it is a potential target for drug or vaccine design. Vaccine design could be in the form of a recombinant virus with mutated Nsp1 incapable of down-regulating the interferon response or a live-attenuated virus. Liu et al. (2022) have shown that a single intranasal delivery of an attenuated SARS-CoV-2, which included a pair of mutations in Nsp1, induced both mucosal and systemic IgA and IgG-mediated protection in the Golden Syrian hamster COVID-19 model [3].

There is still much to be learned about the different activities of Nsp1 in different SARS-CoV-2 variants of concern. This study supports previous observations that deletions in Nsp1 of coronaviruses are attenuating, as demonstrated by a lack of weight loss and clinical signs in hamsters infected with BA.4. While it is reasonable to assume that the deletion in BA.4 Nsp1 is responsible for the observed attenuation in this study, a confirmatory study in which the deletion in BA.4 was genetically introduced into BA.5.2.1 and a hamster challenge study were performed would test this hypothesis conclusively. Further work to support this hypothesis may lead to a better understanding of the drivers of virulence and molecular targets for anti-viral therapy and the rational design of future mucosal, live-attenuated vaccines.

## Figures and Tables

**Table 1:** Geometric mean of neutralizing antibody titers from BA.4 and BA.5.2.1-infected hamsters at 7 and 28 days post-infection with ancestral (VIC01), BA.4 and BA.5.2.1 viruses.

Days Post-Infection	Challenge Virus	Mean Neutralizing Antibody Titer (ND_50_)		
		VIC01	BA.4	BA.5
**7**	**BA.4**	187	14,471	9446
	**BA.5**	967	67,363	51,209
**28**	**BA.4**	2144	1008	1084
	**BA.5**	3709	7007	7970

**Table 2:** Geometric mean of neutralizing antibody titers from BA.1, BA.4, and BA.5.2.1 convalescent hamster sera against BQ.1.22 and XBB.1.1.

Infection Virus		Geometric Mean Neutralizing Antibody Titre (ND_50_)			
	BA.1	BA.4	BA.5.2.1	BQ.1.22	XBB.1.1
**BA.1**	373			≤20	≤20
**BA.4**		1008		295	≤20
**BA.5.2.1**			7970	1078	112

## Data Availability

Not applicable.

## Supplementary Materials

The following supporting information can be downloaded at: https://www.mdpi.com/article/10.3390/v15051133/s1, Supplementary Data File S1: Sequence data of the virus banks; Figure S1: Assessment of live viral titer in throat swabs (2 DPI); Figure S2: Assessment of viral shedding from the respiratory tract and viral load in the lung against earlier Omicron sub-lineages and ancestral virus VIC01; Figure S3: Comparison histopathological analysis of the nasal cavity and lung 7 days post-infection against previous Omicron sub-lineages and ancestral viruses.
