# Supplementary material for: The Omicron Sub-Variant BA.4 Displays a Remarkable Lack of Clinical Signs in a Golden Syrian Hamster Model of SARS-CoV-2 Infection
Source: Viruses. 2023 May 10;15(5):1133. doi: 10.3390/v15051133 (PMC10224153; doi:10.3390/v15051133)
Supplement: Supplementary file 1 [file viruses-15-01133-s001.zip › viruses-2384220-supplementary.pdf]

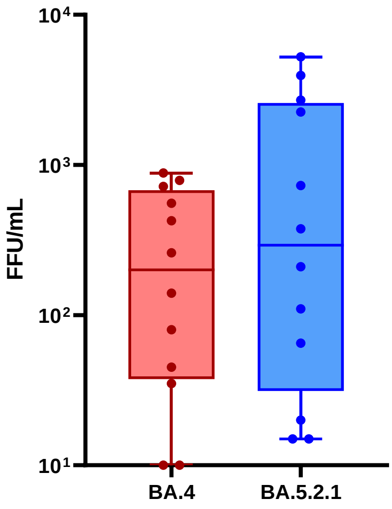

Supplementary Figure S1: Assessment of live viral titer in throat swabs 2 DPI. A focus formation assay was performed on throat swabs from 2 days post infection. Box plots show medians and 25th to 75th percentiles, and whiskers represent minimum and maximum values; all data points are shown. Statistical analyses performed by unpaired t test showed no significant difference.

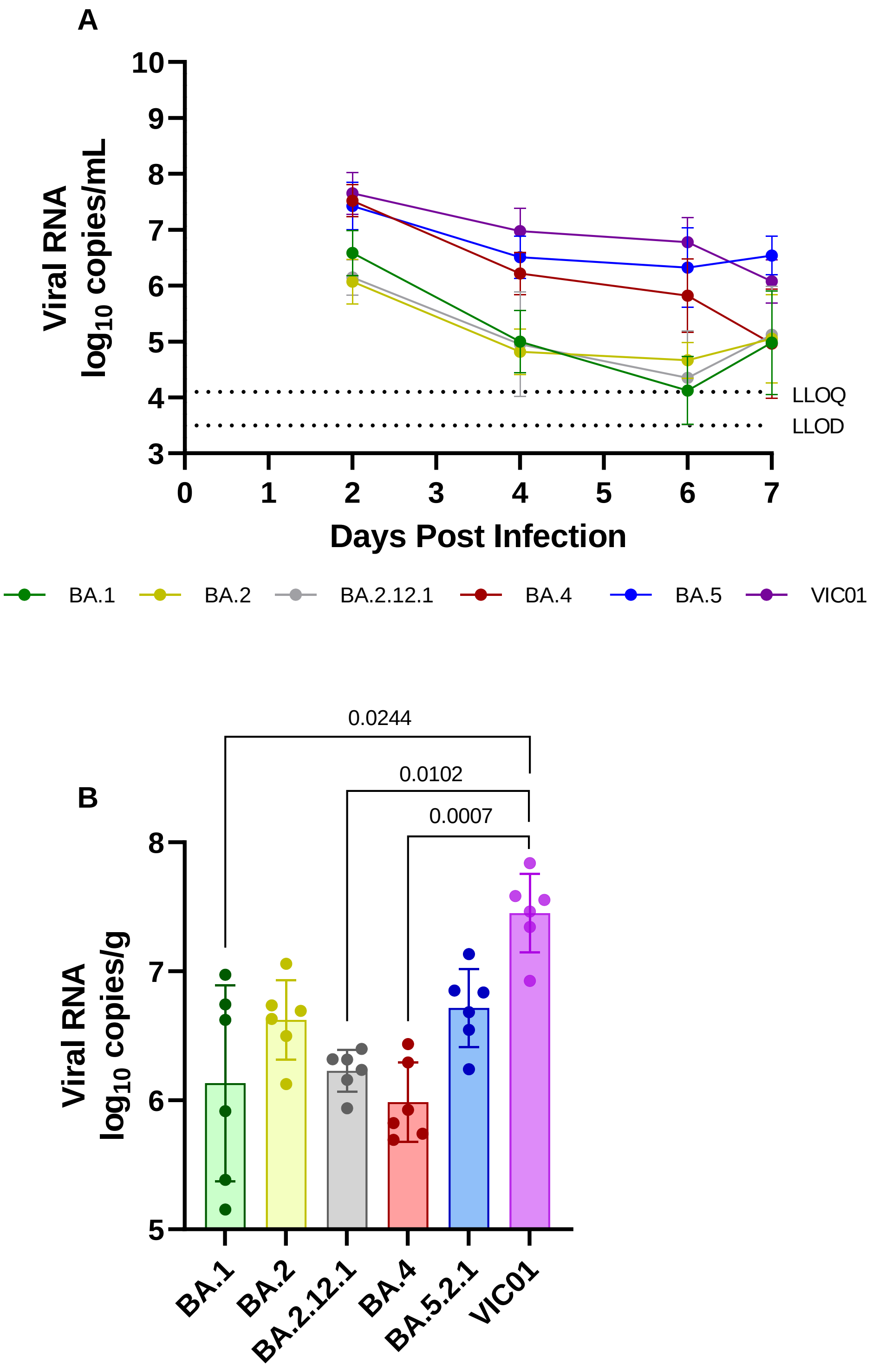

Supplementary Figure S2: Assessment of viral shedding from respiratory tract and viral load in the lung against earlier Omicron sub-lineages and ancestral virus VIC01. (**A**) Time-course of viral shedding from the upper respiratory tract. (**B**) viral load in the lung at 7 days post infection. Graphs shows geometric mean and SD. Statistical analyses were performed using unpaired t test with Welch's correction on log_{10} transformed data.

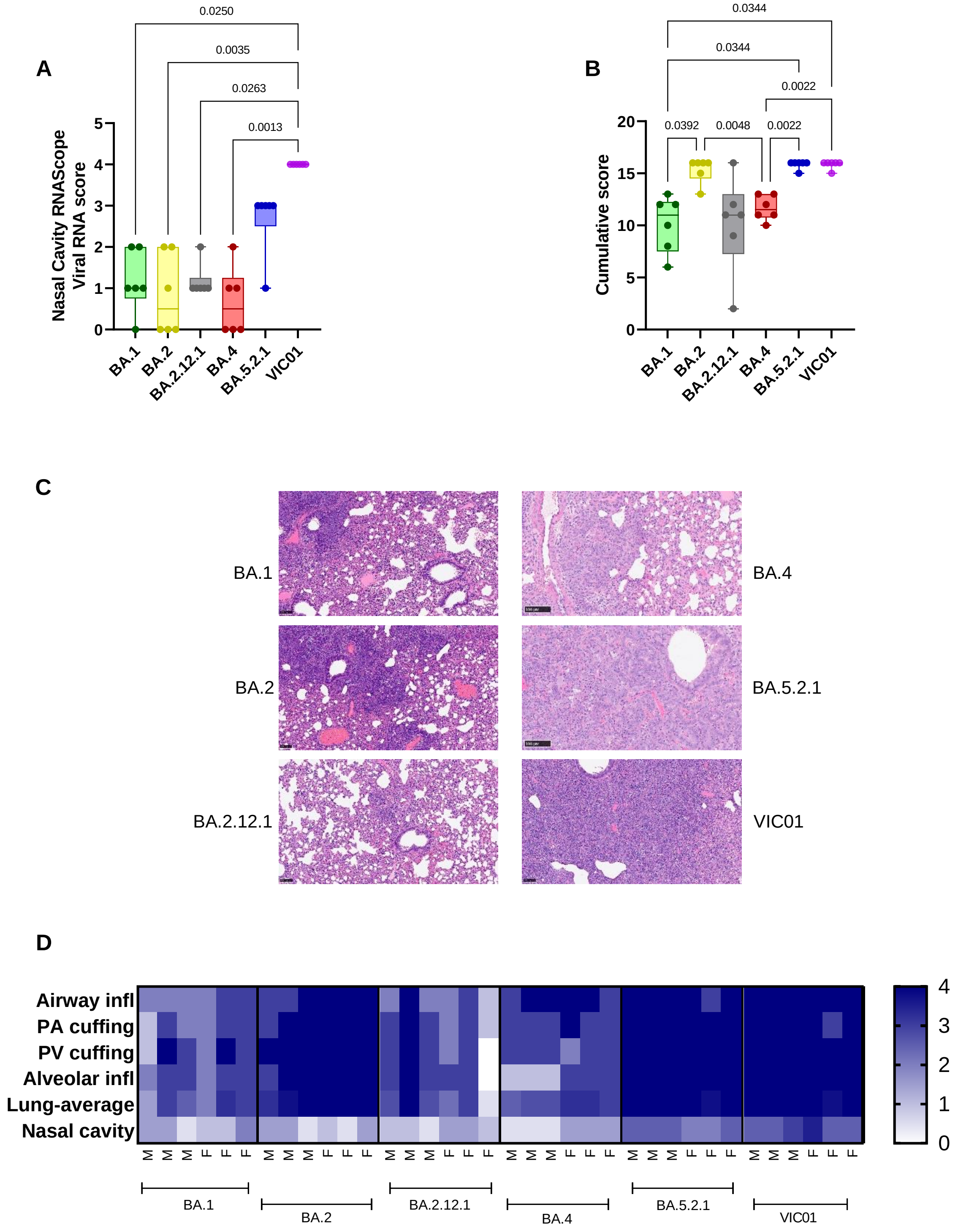

Supplementary Figure S3: Comparison histopathological analysis of nasal cavity and lung 7 days post infection to previous Omicron sub-lineages and ancestral virus. (**A**) Subjective scores of the presence of SARS-CoV-2 RNA in the nasal cavity measured by ISH. (**B**) Cumulative lung histopathology score, determined by image analysis (Nikon-NIS-Ar). Box plots show medians and 25th to 75th percentiles, and whiskers represent minimum and maximum values; all data points are shown. Statistical analyses performed using Kruskal Wallis test with Dunn's multiple comparison. (**C**) Representative images of

lung H&E (bar represents 100µm). (**D**) Heatmap to show comparison of the severity of the lung lesions in the cranial and caudal lung lobe tissue sections.
